# Differences in olfactory functional connectivity in early-onset depression and late-onset depression

**DOI:** 10.1093/psyrad/kkad030

**Published:** 2023-12-04

**Authors:** Ben Chen, Mingfeng Yang, Meiling Liu, Qiang Wang, Huarong Zhou, Min Zhang, Le Hou, Zhangying Wu, Si Zhang, Gaohong Lin, Xiaomei Zhong, Yuping Ning

**Affiliations:** Geriatric Neuroscience Center, The Affiliated Brain Hospital of Guangzhou Medical University, Guangzhou, Guangdong Province 510370, China; Smell & Taste Clinic, Department of Otorhinolaryngology, TU Dresden 01307, Germany; Geriatric Neuroscience Center, The Affiliated Brain Hospital of Guangzhou Medical University, Guangzhou, Guangdong Province 510370, China; Geriatric Neuroscience Center, The Affiliated Brain Hospital of Guangzhou Medical University, Guangzhou, Guangdong Province 510370, China; Geriatric Neuroscience Center, The Affiliated Brain Hospital of Guangzhou Medical University, Guangzhou, Guangdong Province 510370, China; Geriatric Neuroscience Center, The Affiliated Brain Hospital of Guangzhou Medical University, Guangzhou, Guangdong Province 510370, China; Geriatric Neuroscience Center, The Affiliated Brain Hospital of Guangzhou Medical University, Guangzhou, Guangdong Province 510370, China; Geriatric Neuroscience Center, The Affiliated Brain Hospital of Guangzhou Medical University, Guangzhou, Guangdong Province 510370, China; Geriatric Neuroscience Center, The Affiliated Brain Hospital of Guangzhou Medical University, Guangzhou, Guangdong Province 510370, China; Geriatric Neuroscience Center, The Affiliated Brain Hospital of Guangzhou Medical University, Guangzhou, Guangdong Province 510370, China; Geriatric Neuroscience Center, The Affiliated Brain Hospital of Guangzhou Medical University, Guangzhou, Guangdong Province 510370, China; Geriatric Neuroscience Center, The Affiliated Brain Hospital of Guangzhou Medical University, Guangzhou, Guangdong Province 510370, China; Geriatric Neuroscience Center, The Affiliated Brain Hospital of Guangzhou Medical University, Guangzhou, Guangdong Province 510370, China; The first School of Clinical Medicine, Southern Medical University, Guangzhou, Guangdong Province 510515, China; Guangdong Engineering Technology Research Center for Translational Medicine of Mental Disorders, Guangzhou 510370, China; Key Laboratory of Neurogenetics and Channelopathies of Guangdong Province and the Ministry of Education of China, Guangzhou Medical University, Guangzhou 510370, China

**Keywords:** late-life depression, olfactory dysfunction, MRI, functional connectivity, late-onset depression, resting-state

## Abstract

**Background:**

Late-onset depression (LOD) and early-onset depression (EOD) exhibit different pathological mechanisms and clinical phenotypes, including different extents of olfactory dysfunction. However, the brain abnormalities underlying the differences in olfactory dysfunction between EOD and LOD remain unclear.

**Objective:**

The aim of this study was to compare the functional connectivity (FC) patterns of olfactory regions between EOD patients and LOD patients and examine their relationship with cognitive function.

**Methods:**

One hundred and five patients with EOD, 101 patients with LOD and 160 normal controls (NCs) were recruited for the present study. Participants underwent clinical assessment, olfactory testing, cognitive assessments, and magnetic resonance imaging. Eight regions of the primary and secondary olfactory regions were selected to investigate olfactory FC.

**Results:**

Patients with LOD exhibited decreased odor identification (OI) compared with patients with EOD and NCs. The LOD group exhibited decreased FC compared with the EOD and NC groups when primary and secondary olfactory regions were selected as the regions of interest (the piriform cortex, lateral entorhinal cortex, and orbital-frontal cortex). Additionally, these abnormal olfactory FCs were associated with decreased cognitive function scores and OI, and the FC between the left orbital-frontal cortex and left amygdala was a partial mediator of the relationship between global cognitive scores and OI.

**Conclusion:**

Overall, patients with LOD exhibited decreased FC in both the primary and secondary olfactory cortices compared with patients with EOD, and abnormal olfactory FC was associated with OI dysfunction and cognitive impairment. The FC between the orbital-frontal cortex and amygdala mediated the relationship between global cognitive function and OI.

## Introduction

Late-life depression (LLD) is one of the major risks of disability and dementia in the geriatric population, and it affects 3.68–4.60% of elderly people each year (Invernizzi *et al*., 2021). An increasing number of studies have suggested that people with late-onset depression (LOD) (the first depressive episode occurs after age 60) and early-onset depression (EOD) (the first depressive episode occurs before age 60) exhibit different responses to antidepressant and electroconvulsive therapy (Dols *et al*., 2017), and the risk of developing dementia is higher in patients with LOD than in patients with EOD (Lee *et al*., 2021), which may result from the various pathological mechanisms of the two disorders. Specifically, LOD is more closely related to neurodegeneration, microvascular dysfunction, stroke, and other pathological aging processes, whereas EOD is more strongly associated with genetic susceptibility and adverse life events (Choi *et al*., 2017; Paranthaman *et al*., 2012). Compared with patients with EOD, patients with LOD exhibited slower information processing speed, poorer executive function (Cheng *et al*., 2020), nerve growth factor deficiency (Shi *et al*., 2010), higher inflammatory levels (Perna *et al*., 2022), increased cortical Aβ burden (Loureiro *et al*., 2020), more hippocampal atrophy (Ballmaier *et al*., 2008, Gao *et al*., 2022), more white matter hypersignal (Choi *et al*., 2017), and different modular organization of the functional brain network (Mai *et al*., 2021).

Aging is a risk factor for olfactory system deterioration, and a meta-analysis indicated that olfactory dysfunction starts in the fifth decade of life in healthy people (Zhang and Wang, 2017). Odor identification (OI) is a predictor of Alzheimer's disease (AD) because it represents early pathological changes in the entorhinal cortex and hippocampus (Lafaille-Magnan *et al*., 2017), and its predictive effect has been shown in amnestic mild cognitive impairment (MCI) patients and community elderly adults (Murphy, 2019). Furthermore, our previous studies indicate that OI dysfunction may contribute to the risk of developing dementia in patients with LLD, indicating that there are more severe impairments in cognitive function and the ability to perform activities of daily living, and suggesting more severe structural and functional brain abnormalities in LLD patients with OI dysfunction than in those with normal OI (Chen *et al*., 2018; Chen *et al*., 2021). Interestingly, our previous study also suggested that the olfactory processing of patients with EOD and LOD may be different, that LOD patients exhibit worse OI than EOD patients, and that the variations in OI among patients can be attributed to differences in their memory and language function (Liu *et al*., 2022). Therefore, this evidence indicates that there may be an interactive effect between LLD and age of first depressive episode on OI, and this effect should be considered when using OI to predict dementia risk in LLD patients. However, the pathological mechanism underlying the different OIs between EOD and LOD remains unclear.

OI processes include peripheral perception of the primary olfactory cortex (such as the piriform cortex) and high-order processing of the secondary olfactory cortex [such as the orbital-frontal cortex (OFC) and insular cortex] (Rai *et al*., 2021). Because the olfactory pathway strongly overlaps with the cognitive map (Murphy, 2019), disrupted activity and functional connectivity (FC, the statistical correlation or synchronization of neural activity between brain regions that provides insights into their functional relationships and communication) of the olfactory regions are associated with not only OI dysfunction but also cognitive impairment (Chen *et al*., 2022; Lu *et al*., 2019). Additionally, our previous studies suggested that LLD patients exhibited disrupted olfactory FC compared with healthy controls. Furthermore, abnormal FCs of olfactory regions are associated with OI, global cognition, and language function in patients with LLD (Yang *et al*., 2022). However, it remains unclear whether abnormal olfactory FC is involved in the differences in OIs and cognitive functions between EOD and LOD.

Based on this evidence, we hypothesized that patients with LOD exhibit decreased FC in both primary and secondary olfactory regions compared with patients with EOD, and this decreased FC may be involved in the difference in OI and cognitive function between patients with EOD and those with LOD. Therefore, the present study aimed to (i) compare olfactory FC patterns between EOD and LOD patients and (ii) explore the relationship among olfactory FC, OI dysfunction, and cognitive impairment in EOD and LOD patients. The present study provides an in-depth investigation of the different pathological mechanisms between EOD and LOD and contributes to a reasonable application of OI for predicting dementia risk in patients with LLD.

## Materials and methods

### Participants

Two hundred and six patients with LLD were recruited from the Affiliated Brain Hospital of Guangzhou Medical University, and 160 normal controls (NCs) matched for age and gender were recruited from communities in Guangzhou. All participants or relevant legal guardians provided written informed consent to participate in the study. The study protocol and assessments were approved by the Ethics Committees of the Affiliated Brain Hospital of Guangzhou Medical University.

Patients with LLD were included in the present study according to the following inclusion criteria: (i) age >55 years, (ii) major depression diagnosed according to DSM-IV criteria, and (iii) clinical staging and diagnosis made by at least two neurologists with expertise in dementia, a neuropsychologist, and a geriatric psychiatrist. NCs were included if they (1) were right-handed, (ii) exhibited normal cognitive function, and (iii) had no past history of depression. The exclusion criteria for both LLD patients and NCs were as follows: (i) a history of other major psychiatric disorders, such as bipolar disorder or schizophrenia; (ii) physical illnesses, such as anaemia or hypothyroidism, that could lead to depressive episodes; (iii) neurological disorders, such as brain tumours, Parkinson's disease, multiple sclerosis, and stroke; (iv) current or previous psychiatric symptoms; (v) head injury with loss of consciousness >30 min; and (vi) other conditions significantly affecting the sense of smell, including active upper airway/sinus infection or dyspnoea at the time of testing, traumatic or congenital olfactory impairment (known nasal tumours or polyps), current or recent (in the last 6 months) smoking, and alcohol abuse or substance dependence. For further analyses, patients with LLD were divided into two subgroups: the EOD group, whose first depressive episode occurred before age 60, and the LOD group, whose depressive episode occurred after the age of 60.

### Neuropsychological assessments

Participants underwent comprehensive neuropsychological testing to evaluate various domains of cognition after completing a standard clinical assessment. (i) The Mini-Mental State Examination (MMSE) (score range 0–30) was used to assess global cognitive function (Tombaugh and McIntyre, 1992); (ii) the Auditory Verbal Learning Test (AVLT) N5 was used to assess memory (score range 0–12) (Hawkins *et al*., 2004); (iii) the Trail-Making Test B was used to assess executive function with scores depending on the number of seconds it took for the participant to complete the test (Tombaugh, 2004); (iv) the Boston Naming Test (BNT) was used to assess language function with scores ranging from 0–30 (Bezdicek *et al*., 2021); and (v) the Symbolic Digit Transformation Test (SDMT) was used to assess information processing speed, with scores depending on the number of symbols recorded correctly within 90 s (Fellows and Schmitter-Edgecombe, 2019). Additionally, the 17-item Hamilton Depression Rating Scale (HAMD-17) was used to evaluate the severity of depression (Schwab *et al*., 1967). All scale assessments were completed by two trained professional psychiatrists who passed a concordance assessment.

### Olfactory assessments

Before the olfactory test, participants were asked to complete a questionnaire that excluded factors that could affect olfactory function (i.e. history of nasal trauma and related surgery, history of radiation or chemotherapy, nasal congestion, etc.). OI was assessed using the Sniffin’ Sticks Screen 16 test for olfactory assessment (Perna *et al*., 2022). Participants were asked to smell 16 common odors in a specific order and were asked to select one image out of four that best matched the odor smelled; only one of the four images was correct. Participants were given scores ranging from 0 to 16. The experiment was conducted in a quiet, well-ventilated room free of odors. All participants who completed the neuropsychological assessment were given the OI test on the same day.

### MRI data acquisition and processing

Participants underwent magnetic resonance imaging (MRI) scans after the neuropsychological assessments. A Philips 3.0 T MR system (Achieva, Netherlands) at The Affiliated Brain Hospital of Guangzhou Medical University was used to acquire the imaging data. For each participant, an anatomical image was obtained with a sagittal 3D gradient echo. Sagittal resting-state functional MRI (fMRI) datasets of the whole brain were obtained in 8 min with a single-shot gradient echo-planar imaging pulse sequence. The resting-state fMRI scanning parameters were as follows: echo time (TE) = 30 ms; repetition time (TR) = 2000 ms; flip angle = 90°; number of slices = 33; slice thickness = 4 mm; matrix size = 64 × 64; and field of view = 220 × 220 mm.

The preprocessing of resting-state fMRI datasets was carried out using the Data Processing Assistant for Resting-State 5.1 (Yan *et al*., 2016). The first 10 volumes were discarded to ensure steady-state data. The remaining 230 images were corrected for timing differences and for head motion. A record of participants’ head motions was provided after realignment correction. Participants who had >2 mm maximum displacement in any plane, 2° of angular motion, and 0.2 mm mean framewise displacement were excluded from further analysis. To minimize the influence of head motion, the mean framewise displacement of each participant was regressed out in the group-level analysis. Then, the motion-corrected images were spatially normalized into the standard Montreal Neurological Institute (MNI) Echo Planar Imaging template, resliced to a voxel size of 3 × 3 × 3 mm^3^ resolution, and smoothed using a 6 mm full-width at half-maximum Gaussian kernel. Linear trends were removed from the data, and nuisance covariates were then regressed out from each time series, including signals indicating white matter and cerebrospinal fluid as well as the Friston-24 parameters of head motion. To reduce the effect of low-frequency drifts and high-frequency noise, a bandpass filter (0.01 Hz < *f* < 0.1 Hz) was applied.

### Seed-based FC analysis

Regions of interest (ROI) with a radius of 6 mm centred around eight MNI coordinates were selected. The regions included the left and right piriform cortices [(−22, 0, 14), (22, 2, 12)], the left and right OFC [(−24, 30, 10), (28, 34, 12)], and the left and right insula [(−30, 18, 6), (28, 16, 8)]; these regions were obtained from a meta-analysis that identified these three left and right brain regions as those most likely to be activated by olfactory stimulation (Seubert *et al*., 2013). In addition, the lateral entorhinal cortex (LEC) was selected according to the mask reported in the study by Maass et al.(Maass *et al*., 2015). The time courses of all voxels within each ROI were extracted and averaged as the seed point reference time courses. Individual-level FC maps of the eight ROI were obtained by calculating Pearson's correlation coefficients between the mean time courses of the ROI and the time courses of each voxel in the whole brain. Then, Fisher's *r*-to-*z* transformation was performed to improve normality. The *z* score FC maps were used for group-level statistical analyses.

### Statistics

Statistical Package for the Social Sciences version 25.0 (IBM SPSS v.25.0) was used to perform the statistical analyses. The results were visualized with GraphPad Prism v.9.0.0 and the BrainNet Viewer (http://www.nitrc.org/projects/bnv/) (Xia *et al*., 2013). The neuropsychological differences among participants were evaluated by one-way analysis of covariance (ANCOVA). *Post hoc* comparisons were made using the *post hoc* least significant difference test. ANCOVA was used to assess the *z* score maps for each ROI to detect significant differences among the three groups. Gaussian random field theory was used for multiple comparison correction (voxel *P* value < 0.001; cluster *P* < 0.05). The mean *z* scores of significantly different regions among groups in ANCOVA were extracted for *post hoc* analysis (significance level 0.05, least significant difference correction). Age, gender, HAMD scores, and framewise displacement were chosen as covariates. The associations between OI and cognitive test scores were evaluated by partial correlation analyses. Stepwise multiple linear regression was used to analyse the effects of different FCs on OI scores and the effects of various cognitive scores on different FCs. Mediation analyses were performed to investigate the potential relationship between cognitive score (independent variable) and OI (dependent variable). FC values were regarded as mediators, and we calculated the mediation model in PROCESS v.3.4 running in an SPSS v.25 environment. The level of confidence for all confidence intervals in the output was 95% with 5000 bootstrap samples. Effects were considered significant if 0 was not contained in the 95% confidence intervals. Age, gender, and HAMD scores were chosen as covariates in the partial correlation, regression, and mediation analyses.

## Results

### Demographic information, OI, and cognitive function

There was no significant difference in age (LLD 67.4 ± 6.2, NC 67.1 ± 6.0, *t* = 0.305, *P* = 0.761) or gender distribution (men/women) (LLD 51/155, NC 40/120, *χ*² = 0.003, *P* = 0.958) between the LLD and NC groups. A total of 105 patients satisfied the EOD criteria, and 101 patients satisfied the LOD criteria.

When LLD patients were divided into the EOD group and LOD group, there were significant differences in the HAMD, OI, MMSE, AVLT N5, TMTB, BNT, and SDMT scores between the EOD group and the NC group and between the LOD group and the NC group and the NC group (*P* < 0.05); no significant difference was found in TMTB scores among the three groups (*P* > 0.05). In the *post hoc* analyses, both the EOD group and LOD group exhibited higher HAMD scores and lower MMSE, AVLT N5, and SDMT scores than the NC group. Additionally, the LOD group exhibited lower OI and BNT scores than the NC and EOD groups. No significant difference in HAMD scores was found between the EOD group and LOD group (*P* > 0.05) (Table 1).

**Table 1:** Differences in OI and neuropsychological scores between EOD and LOD groups.

	NC (*n* = 160)	EOD (*n* = 105)	LOD (*n* = 101)	*F*	*Post hoc*
HAMD	2.7 ± 3.1	10.9 ± 8.0	12.3 ± 8.7	37.729***	A < B, C
OI	10.8 ± 2.2	10.8 ± 2.6	9.2 ± 2.5	10.845***	A, B > C
MMSE	25.7 ± 2.9	23.4 ± 4.5	22.0 ± 5.4	28.794***	A > B, C
AVLT N5	5.4 ± 2.9	4.4 ± 2.9	4.0 ± 3.0	18.761***	A > B, C
TMTB	77.4 ± 40.2	81.3 ± 39.1	97.2 ± 44.4	17.056***	NS
BNT	21.1 ± 3.5	20.4 ± 3.9	18.4 ± 4.9	23.959***	A, B > C
SDMT	32.8 ± 11.2	28.1 ± 11.3	22.7 ± 13.4	25.414***	A > B, C

Abbreviations: TMT-B, Trail-Making Test B; NS: not significant; ***P < 0.001.

### Differences in olfactory FC among the EOD, LOD, and NC groups

**Figure 1:**
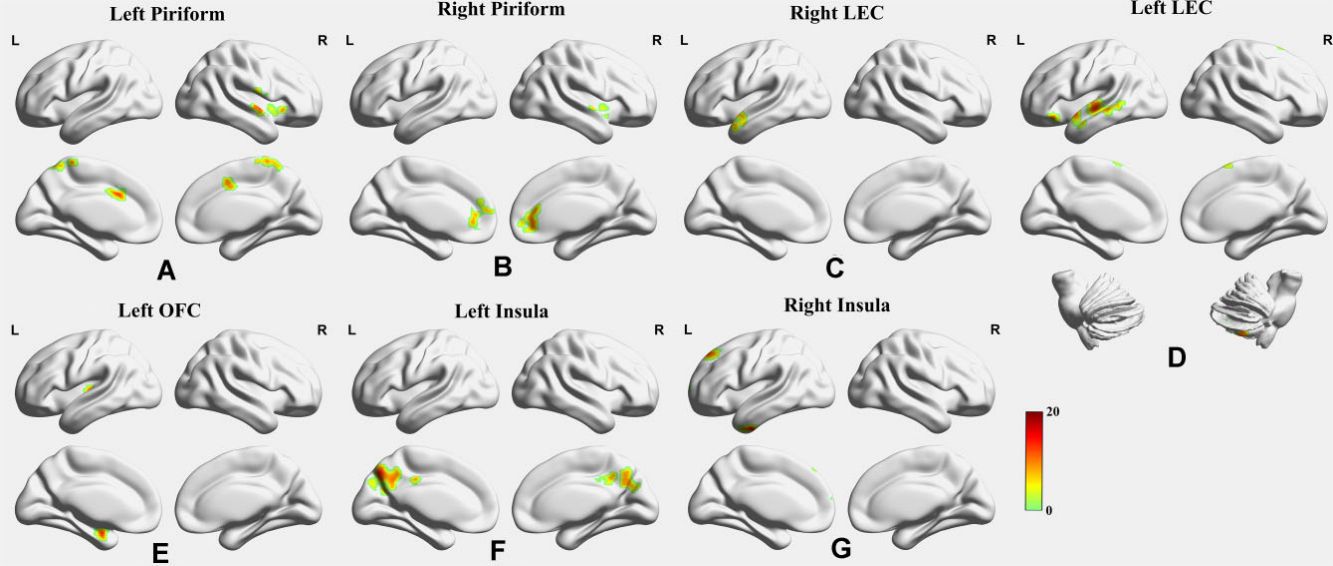
Differences in FC among the EOD, LOD, and NC groups. Differences in FC were found (**A**) between the left piriform cortex and right superior temporal gyrus, the left piriform cortex and left middle cingulum, and the left piriform cortex and right praecuneus; (**B**) between the right piriform cortex and right temporal pole, and between the right piriform cortex and right anterior cingulum; (**C**) between the right LEC FC and left temporal pole; (**D**) between the left LEC and right inferior cerebellum, left LEC and left temporal pole, left LEC and left middle temporal gyrus, and left LEC and left supplementary motor area; (**E**) between the left OFC and left amygdala, and between the left OFC and left Heschl's gyrus; (**F**) between the left insula and left praecuneus; and (**G**) between the right insula and left inferior temporal gyrus, between the right insula and left medial superior frontal gyrus, and between the right insula and left superior frontal gyrus. The color bar represents *F* values in ANCOVA.

In total, 88 health NC participants, 82 LOD patients, and 50 EOD patients agreed to undergo the neuroimaging assessments. In the ANCOVA of FC, significant differences were found among the EOD, LOD, and NC groups (Table 2 and Fig. 1), including FC (i) between the left piriform cortex and right superior temporal gyrus, left piriform cortex and left middle cingulum, and left piriform cortex and right praecuneus; (ii) between the right piriform cortex and right temporal pole and between the right piriform cortex and right anterior cingulum; (iii) between the left LEC and right inferior cerebellum, left LEC and left temporal pole, left LEC and left middle temporal gyrus, and left LEC and left supplementary motor area; (iv) between the right LEC and left temporal pole; (v) between the left OFC and left amygdala and between the left OFC and left Heschl's gyrus; (vi) between the left insula and left praecuneus; and (vii) between the right insula and left inferior temporal gyrus, right insula and left medial superior frontal gyrus, and right insula and left superior frontal gyrus. When the right OFC was selected as the seed, no significant difference was found among the EOD, LOD, and NC groups.

**Table 2:** Differences in FC among the EOD, LOD, and NC groups.

ROI	Brain regions	Peak MNI coordinate			Cluster size	Peak intensity
		*x*	*y*	*z*		
Left piriform cortex						
	Right superior temporal gyrus	57	−3	0	279	13.53
	Left middle cingulum	−6	15	33	120	13.80
	Right precuneus	3	−45	66	117	11.50
Right piriform cortex						
	Right temporal pole	54	15	−12	123	12.11
	Right anterior cingulum	9	39	0	229	11.80
Left LEC						
	Right inferior cerebellum	30	−60	−51	104	12.41
	Left temporal pole	−57	9	−12	158	13.65
	Left middle temporal gyrus	−63	−48	6	187	13.59
	Left supplementary motor area	−3	9	69	81	14.65
Right LEC						
	Left temporal pole	−54	9	−9	67	11.73
Left OFC						
	Left amygdala	−27	−3	−24	39	13.98
	Left Heschl's gyrus	−45	−18	6	43	12.86
Left insula						
	Left precuneus	−9	−69	45	521	14.26
Right insula						
	Left inferior temporal gyrus	−48	−6	−39	38	16.23
	Left medial superior frontal gyrus	−3	66	15	56	11.68
	Left superior frontal gyrus	−15	42	48	73	12.62

**Figure 2:**
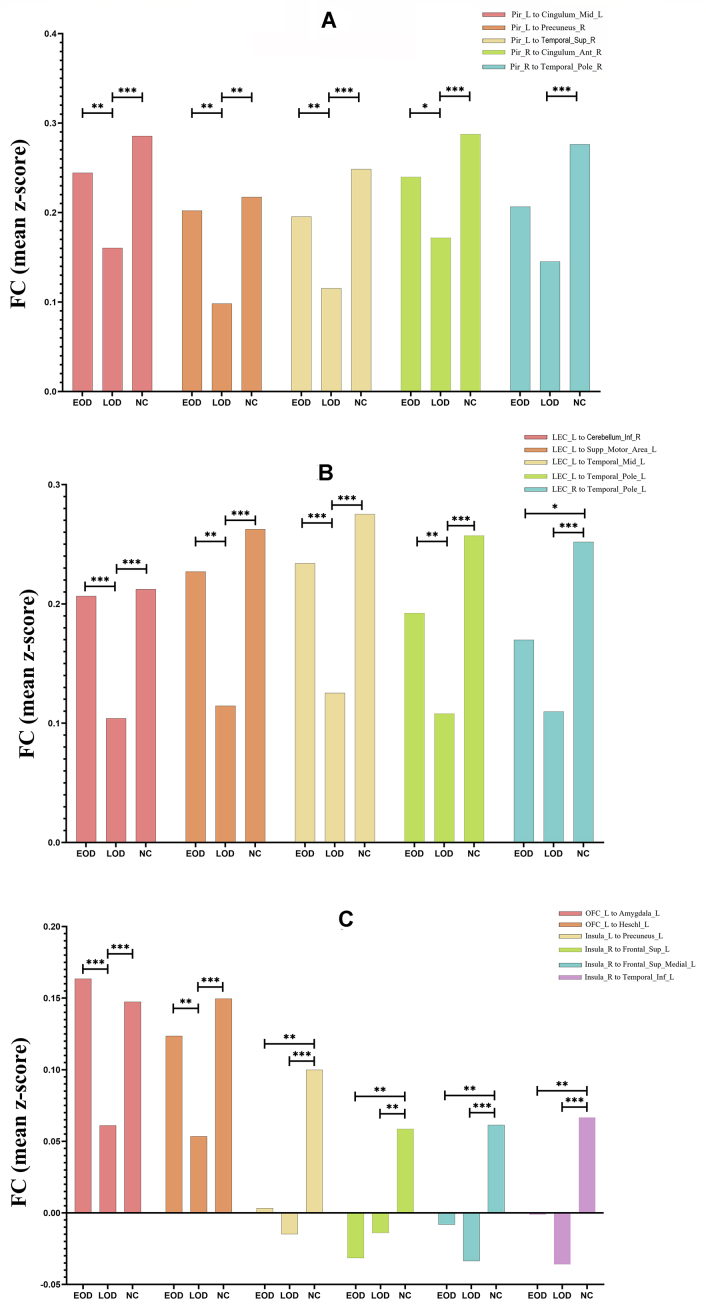
Comparison of FC among the EOD, LOD, and NC groups. *P < 0.05; **P < 0.01; ***P < 0.001. (**A**) The LOD group exhibited significantly decreased FC values compared with the EOD group and NC group when the bilateral piriform cortex was selected as the seed. (**B**) The LOD group exhibited significantly decreased FC values compared with the EOD group and NC group when the bilateral LEC was selected as the seed. (**C**) The LOD group exhibited significantly decreased FC values compared with the EOD group and NC group when the left OFC was selected as the seed, and both the EOD and LOD groups exhibited significantly decreased FC values compared with the NC group when the left and right insula were selected as the seeds. Abbreviations: Pir: piriform gyrus. Ant: anterior, Inf: inferior, Mid: middle, Sup: superior, Supp: supplementary, L: left, R: right.

In the *post hoc* comparison, the LOD group exhibited significantly decreased FC values compared with the EOD group and NC group when the bilateral piriform cortex, bilateral LEC, and left OFC were selected as the seeds. Additionally, both the EOD and LOD groups exhibited significantly decreased FC values compared with the NC group when the left and right insula were selected as the seeds (Fig. 2).

### Association between olfactory FC and OI

Partial correlation analyses suggested that OI was associated with FC between the left piriform cortex and left middle cingulum (*r* = 0.182, *P* = 0.012), FC between the left piriform cortex and right superior temporal gyrus (*r* = 0.153, *P* = 0.035), FC between the right piriform cortex and right anterior cingulum (*r* = 0.147, *P* = 0.043), FC between the left OFC and left amygdala (*r* = 0.239, *P* = 0.001), FC between the right insula and left medial superior frontal gyrus (*r* = 0.238, *P* = 0.001), FC between the right insula and left inferior temporal gyrus (*r* = 0.182, *P* = 0.012), FC between the left LEC and right inferior cerebellum (*r* = 0.156, *P* = 0.032), and FC between the left LEC and left middle temporal gyrus (*r* = 0.149, *P* = 0.040).

**Figure 3:**
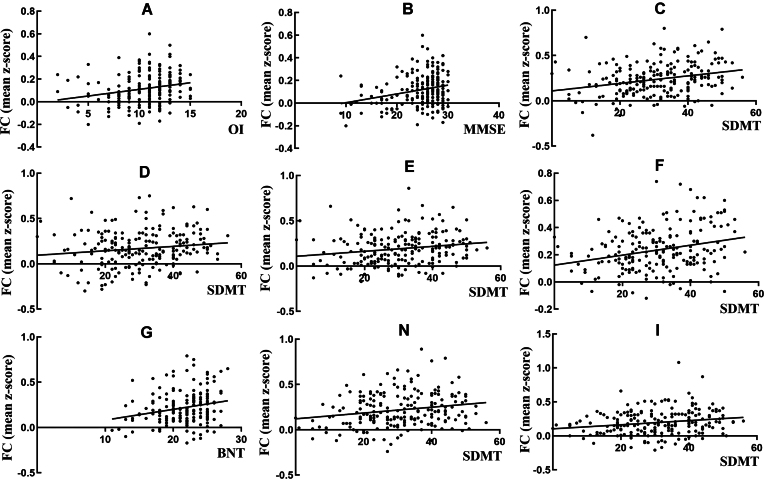
The correlations between FC values and neuropsychological variables in all participants. (**A**) The correlation between OI and FC between the left OFC and left amygdala. (**B**) The correlation between MMSE scores and FC between the left OFC and left amygdala. (**C**) The correlation between SMDT scores and FC between the left piriform and left middle cingulum. (**D**) The correlation between the SMDT scores and FC between the left piriform and right praecuneus. (**E**) The correlation between SMDT scores and FC between the left piriform and right superior temporal gyrus. (**F**) The correlation between SMDT scores and FC between the right piriform and right anterior cingulum. (**G**) The correlation of FC between the right piriform and right temporal poles. (**H**) The correlation between SMDT scores and FC between the left LEC and left middle temporal gyrus. (**I**) The correlation between SMDT scores and FC between the left LEC and left temporal pole.

Regression analyses suggested that OI was most associated with FC between the left OFC and left amygdala (*R*^2^ = 0.145, *β* = 0.223, *t* = 3.314, *P* = 0.001) (Fig. 3A).

### Association between olfactory FC and cognitive function

When various FCs were regarded as the dependent variables and various cognitive scores were designated as the independent variables, the SDMT score was the most highly associated variable in the model of the FC between the left piriform cortex and left middle cingulum, FC between the left piriform cortex and right praecuneus, FC between the left piriform cortex and right superior temporal gyrus, FC between the right piriform cortex and right anterior cingulum, FC between the left LEC and left middle temporal gyrus, and FC between the left LEC and left temporal pole. Additionally, the BNT score was most strongly associated with FC between the right piriform cortex and right temporal pole, and the MMSE score was most strongly associated with FC between the left OFC and left amygdala (Table 3, Fig. 3).

**Table 3:** Association between olfactory FC and cognitive function.

	*R* ^2^	Variables	*β*	*t*	*P*
FC between left piriform cortex and left middle cingulum	0.107	SDMT	0.327	4.510	<0.001
FC between left piriform cortex and right precuneus	0.123	SDMT	0.162	2.243	0.026
FC between left piriform cortex and right superior temporal gyrus	0.159	SDMT	0.212	3.007	0.003
FC between right piriform cortex and right anterior cingulum	0.092	SDMT	0.303	4.147	<0.001
FC between right piriform cortex and right temporal pole	0.066	BNT	3.452	3.452	0.001
FC between left OFC and left amygdala	0.113	MMSE	0.155	2.131	0.035
FC between left OFC and left Heschl's gyrus	NS		NS	NS	NS
FC between left insula and left precuneus	NS		NS	NSNS	NS
FC between left LEC and left middle temporal gyrus	NS		NS	NS	NS
FC between left LEC and left middle temporal gyrus	0.106	SDMT	0.218	2.997	0.003
FC between left LEC and left temporal pole	0.048	SDMT	0.220	2.940	0.004

### Mediating effect of olfactory FC on the relationship between OI and cognition

**Figure 4:**
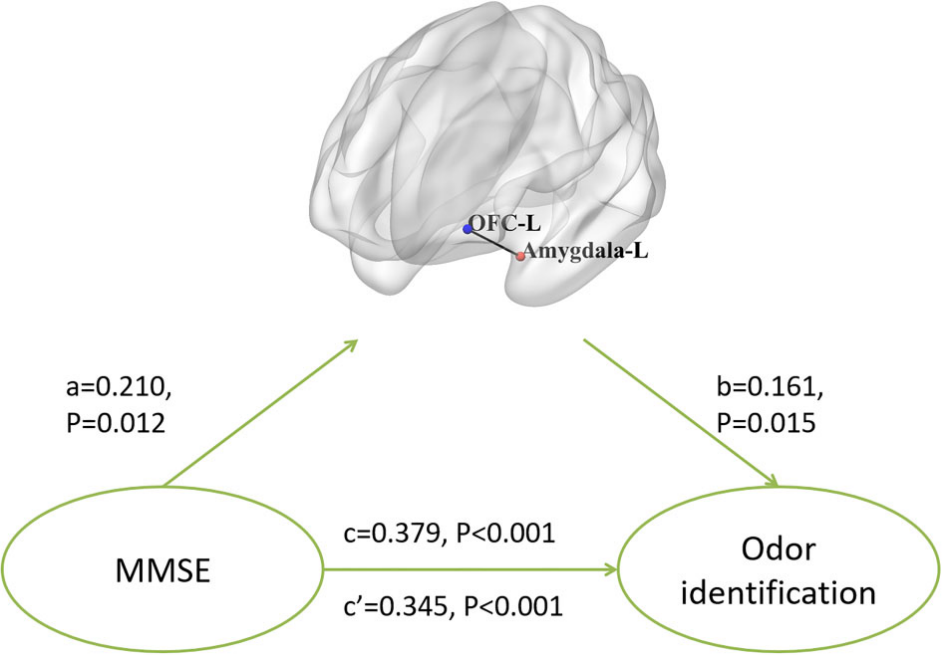
FC between the left OFC and left amygdala mediated the relationship between MMSE scores and OI. a shows the effect of MMSE scores on FC between the left OFC and left amygdala. b shows the effect of FC between the left OFC and left amygdala on OI scores. c′ means the direct effect of MMSE scores on OI scores. c is the total effect of MMSE scores on OI scores.

The total effect of MMSE scores on OI was *β* = 0.379 (*P <* 0.001), and the indirect effect of MMSE scores on OI mediated by the FC between the left OFC and left amygdala was *β* = 0.034 (BootLLCI = 0.002, BootULCI = 0.045). Furthermore, the remaining direct effect of MMSE scores on OI was *β* = 0.345 (*P* < 0.001), with the effect of MMSE scores on FC between the left OFC and left amygdala being *β* = 0.210 (*P* = 0.012) and the effect of FC between the left OFC and left amygdala on OI being *β* = 0.161 (*P* = 0.015). In summary, these results revealed that the FC between the left OFC and left amygdala was a mediator of the relationship between MMSE scores and OI. No significant mediating effect was found in FC values on the association between OI and the other cognitive scores (Fig. 4).

## Discussion and conclusion

The present study compared the OI and olfactory FC of patients with EOD and LOD and explored the relationship among olfactory FC, OI dysfunction, and cognitive impairment. The main results are as follows: (i) the LOD group exhibited worse OI than the EOD group; and (ii) the LOD group exhibited decreased FC compared with the EOD group and NC group when the piriform cortex, LEC, and left OFC were selected as the seeds. Additionally, both the EOD and LOD groups exhibited significantly decreased FC compared with the NC group when the insula cortex was selected as the seed. (iii) Olfactory FCs were associated with OI and cognitive function (especially information processing speed), and FC between the left OFC and left amygdala mediated the relationship between global cognitive function and OI.

LLD accompanied by OI dysfunction and olfactory impairment is associated with more severe cognitive deficits, impairments in activities of daily living, and structural and functional brain damage compared to LLD with intact olfaction, suggesting that individuals with LLD and olfactory impairment may exhibit a higher risk of developing dementia (Chen *et al*., 2018; Chen *et al*., 2021). However, whether the age of the first episode of depression influences OI in patients with LLD has not been fully explained. Consistent with our previous studies, the present study with a larger sample size demonstrated that the OI score was lower in patients with LOD than in patients with EOD and controls, suggesting that abnormalities in olfactory processing are more obvious in patients with LOD (Liu *et al*., 2022). In the cognitive function comparison, both EOD and LOD patients exhibited impaired global cognitive function, information processing speed, and memory, which was consistent with previous results in LLD patients. Interestingly, the language deficit exhibited a similar tendency to OI: it was found in LOD patients but not EOD patients. OI processes not only rely on odor perception but also involve many higher cognitive processes (Dulay *et al*., 2008), particularly in naming (Liu *et al*., 2022). Therefore, the worse OI and naming performance in LOD patients may reflect a more severe dysfunction in overlapping region responses for olfaction and language. Therefore, the current results indicate that there are interactive effects of age and depression on OI, and the age of the first episode of depression needs to be considered when applying OI dysfunction to predict dementia risk in LLD patients.

Similar to the psychophysical results of OI, the LOD group exhibited decreased FC in the primary and secondary olfactory regions compared with the EOD and HC groups, and the olfactory FCs were relatively intact in the EOD group. Additionally, these decreased olfactory FCs were associated with OI dysfunction and cognitive impairment. OI requires not only peripheral perception of odor molecules but also high-order processing of olfactory information (Rai *et al*., 2021), and damage to both the primary and secondary olfactory cortices could lead to OI dysfunction (Chen *et al*., 2022). Considering that the function of many olfactory regions is also responsible for cognitive and emotional processing (Murphy, 2019), the worse OI and olfactory FC in the LOD group suggested that these participants suffered more severe and widespread brain abnormalities associated with cognitive impairment and affective disorders, which is consistent with previous evidence (Ballmaier *et al*., 2008; Choi *et al*., 2017; Mai *et al*., 2021). Additionally, in AD patients, abnormalities in olfactory FC have been found, including disrupted FC between the olfactory network and the right hippocampus (Lu *et al*., 2019) and lower FC between the left OFC and right frontal area and between the right OFC and right temporal area (Lee *et al*., 2020). Interestingly, the present results indicated that the pattern of disrupted olfactory FC in LOD patients was closer to that in AD patients, which is consistent with previous opinions that LOD patients exhibit higher AD risk than EOD patients (Pagni *et al*., 2022) (van Reekum *et al*., 1999).

Among various abnormal olfactory FCs, the disconnection between the OFC and amygdala seems to be the most special occurrence, mediating the relationship between OI and global cognitive function. Both the OFC and amygdala are not only high-order olfactory regions but also centres of emotional processing (Zald and Pardo, 1997). A growing body of fundamental studies has indicated that there are strong anatomical connections between the OFC and the amygdala (Stein *et al*., 2007), and disrupted FC of the OFC-amygdala has been found both in resting-state and task-fMRI studies (Liao *et al*., 2010; Sladky *et al*., 2015). Furthermore, changes in brain activity within the left amygdala and the left OFC when receiving odor stimulation were highly intercorrelated (Zald and Pardo, 1997), and the disconnection of the OFC-amygdala was found to be associated with more severe depression and anxiety, suggesting that inefficient top-down modulation from the OFC to the amygdala leads to hyperactivity of fear-processing circuits toward negative or threat cues (Mao *et al*., 2020). Consistently, the current study indicated that the LOD group exhibited subtly higher depressive scores than the EOD group. Moreover, the positive association between this FC and between OI and global cognition suggested that OFC downregulation in the amygdala is also important in olfactory and cognitive processing, and the results of mediation analyses suggested that cognitive impairment may facilitate OI dysfunction by interrupting FC between the OFC and amygdala. However, their causal relationship still needs to be verified by task-fMRI and longitudinal studies.

The piriform cortex is an important part of the primary olfactory cortex, which is responsible for olfactory perception, valence, and action (Chen *et al*., 2022). It receives neuronal projections from the olfactory bulb and is widely connected to secondary olfactory brain regions. Additionally, the piriform cortex is vulnerable to the neurofibrillary tangles and amyloid β seen in patients with AD (Bathini *et al*., 2019; Devanand, 2016). Interestingly, patients with mild AD and amnestic MCI exhibited reduced activation in the right anterior piriform cortex when receiving olfactory stimulation compared to healthy elderly individuals, and odor discrimination was associated with the activation of the right piriform cortex (Kjelvik *et al*., 2020). Furthermore, our previous study suggested that FC between the right piriform cortex and right superior parietal lobule was positively associated with OI scores in patients with LLD, suggesting that decreased FC of the piriform cortex was involved in OI dysfunction in LLD (Yang *et al*., 2022). In the olfactory pathway, the LEC receives olfactory information from the piriform cortex and interacts with the hippocampus to encode and retrieve olfactory memory (Chapuis *et al*., 2013). Notably, the LEC is among the first regions to be affected by tau during AD development, and structural and functional abnormalities in the LEC have been found to be associated with OI dysfunction (Murphy, 2019). In patients with LLD, the disruption of the amygdala-entorhinal-hippocampal network in LLD was found to be involved in emotional and memory processing (Leal *et al*., 2017), but its relationship with OI has not yet been explored. In the present study, both EOD patients and LOD patients exhibited decreased FC of the piriform cortex and LEC, and these disconnections were more obvious in LOD patients. These results indicate the more severe disconnection of the olfactory pathway with other regions in LOD patients and provide evidence of the higher risk of developing AD in LOD patients compared with that of EOD patients.

The insular cortex plays a key role in the integration of multimodal information and in interoceptive and exteroceptive processing (Koeppel *et al*., 2020). Olfactory input is directly transmitted to the insular cortex without passing the thalamus first, and the mid-dorsal insula may be an integrated oral sensory region that plays a critical role in flavour perception (Mazzola *et al*., 2017). Insular dysfunction has been found in patients with hyposmia, and odor-induced responses in the insular cortex were associated with olfactory function (Han *et al*., 2018). Consistently, decreased insular FCs were associated with OI dysfunction in the present study. Nevertheless, there was no significant difference in insular FC between patients with EOD and those with LOD, although both groups exhibited decreased FC compared with the NC group. However, considering that the function of the insula varies among subregions, future studies applying a more detailed atlas could provide a clearer understanding of insular FC in patients with EOD and LOD.

There were several limitations in the present study. First, the ROI for FC analyses were obtained from a meta-analysis and the LEC mask with high-solution, and these ROI were regarded as most likely to be activated by olfactory stimulation. However, there may be other olfactory regions exhibiting different FCs between patients with EOD and those with LOD, and future studies including more ROI need to be further explored. Second, odor threshold and odor discrimination were not assessed in the present study, and future studies are needed to explore their relationships with olfactory FC. Third, FC was calculated by the association between two different regions, and future studies using analyses of dynamic causal models and Granger causality analysis or using olfactory task-fMRI could further clarify the direction of the abnormal connectivity in olfactory regions. Finally, LLD patients take various types of antidepressant in variable doses, which may be a confounding factor in the FC analyses.

## Conclusion

Patients with LOD exhibited decreased FC in both the primary and secondary olfactory cortex compared with patients with EOD. Abnormal olfactory FC was associated with OI dysfunction and cognitive impairment, and the FC between the OFC and amygdala mediated the relationship between global cognitive function and OI. The present study provides an in-depth understanding of the different pathological mechanisms between EOD and LOD, and emphasizes that the age of the first episode of depression needs to be considered when using OI dysfunction as a predictor of dementia risk in LLD patients.
